# Gut Fungal Community Modulates Fat Deposition in Ningxiang Pigs: Species-Specific Regulation via the Glucose–SCFAs Metabolic Axis

**DOI:** 10.3390/ani15131887

**Published:** 2025-06-26

**Authors:** Pengfei Huang, Hanmin Wang, Juan Wang, Zhenrong Qiu, Chunfeng Wang, Han Liu, Qiye Wang, Yali Li, Huansheng Yang

**Affiliations:** College of Life Sciences, Hunan Normal University, Changsha 410114, China

**Keywords:** NX pig, fat deposition, glucose, SCFAs, gut fungal community

## Abstract

Despite limited understanding of gut fungal roles in fat deposition among indigenous pig breeds, a comparative study between high-fat-accumulating Ningxiang (NX) pigs and lean-type Duroc × Landrace × Yorkshire (DLY) pigs reveals a fungal-driven regulatory mechanism. NX pigs exhibited significantly higher fat percentage, elevated serum glucose, and markedly reduced total colonic short-chain fatty acids (SCFAs) compared to DLY pigs (all *p* < 0.001), with butyrate showing the most pronounced decrease. Beta-diversity confirmed distinct fungal communities (*p* = 0.002), where NX pigs were enriched with *Aspergillus* and *Penicillium*, while DLY pigs harbored dominant *Rhodotorula*. Strong correlations were observed: NX-enriched fungi positively correlated with glucose and negatively with SCFAs, whereas *Rhodotorula* strongly associated with SCFAs (*p* < 0.001). FUNGuild analysis linked *Aspergillus*/*Penicillium* to enhanced polysaccharide degradation and glucose bioavailability. The findings propose a gut fungal-mediated “Glucose–SCFAs axis”: NX-enriched fungi elevate glucose (promoting lipogenesis) and suppress SCFAs (reducing butyrate-mediated adipocyte inhibition), whereas *Rhodotorula* in DLY pigs enhances SCFAs-induced lipolysis. Crucially, we demonstrate that fungal modulation primarily drives fat deposition differences between breeds, offering novel probiotics/antifungal strategies for precision swine breeding.

## Simple Summary

This study reveals a gut fungal-mediated mechanism regulating fat deposition in indigenous Chinese pigs. Compared to lean-type Duroc × Landrace × Yorkshire (DLY) pigs, high-fat Ningxiang (NX) pigs showed significantly higher fat deposition and serum glucose, but markedly lower colonic short-chain fatty acids (SCFAs). Distinct fungal communities were observed: NX pigs enriched with *Aspergillus* and *Penicillium* positively correlated with glucose but negatively with SCFAs, while DLY pigs’ dominant *Rhodotorula* strongly correlated with SCFAs. Metabolic analysis linked NX-enriched fungi to enhanced polysaccharide degradation and glucose bioavailability. We propose a fungal-driven “Glucose–SCFAs axis”: NX-associated fungi elevate glucose while suppressing SCFAs. Conversely, *Rhodotorula* in DLY enhances SCFA-induced lipolysis.

## 1. Introduction

Animal breeding significantly contributes to global economies, with pork production accounting for 35% of worldwide meat consumption [1]. The Ningxiang (NX) pig, as an important indigenous pig breed resource in China, is characterized by its remarkable high-fat phenotype. However, the regulatory mechanisms underlying its fat deposition remain incompletely elucidated. In striking contrast, Duroc × Landrace × Yorkshire (DLY) pigs exhibit outstanding lean meat percentage, providing an ideal comparative model for analyzing species differences in lipid metabolism.

Accumulating evidence highlights the critical role of gut microbial metabolites in host energy partitioning. Optimizing fat/lean ratios through microbial interventions could reduce feed costs while meeting consumer preferences for specialty pork [2]. Short-chain fatty acids (SCFAs) have been demonstrated to regulate systemic fat deposition [3,4]. Mechanistic studies reveal that butyrate suppresses fat accumulation by activating G protein-coupled receptor signaling pathways, effectively reducing high-fat diet-induced weight gain [5]. Conversely, propionate promotes lipid oxidative metabolism through PPARγ-dependent pathways, significantly reducing obesity risk [6,7]. Notably, monosaccharides derived from gut microbiota metabolism may influence lipid homeostasis via distinct mechanisms. Excessive glucose tends to be converted into fat storage [8,9], while the hepatic-specific metabolism of fructose markedly enhances lipogenesis and drives abnormal triglyceride accumulation [10].

Current research predominantly focuses on bacterial communities, with limited attention to fungal communities, which constitute only 0.1–5% of the gut microbiota. Emerging evidence suggests that fungal metabolic activities may indirectly modulate host metabolite levels through cross-kingdom interactions. For instance, *Aspergillus* enhance intestinal SCFA levels by promoting substrate hydrolysis through cellulase secretion [11,12], while Rhizopus elevate peripheral blood monosaccharide levels in hosts by decomposing plant-derived polysaccharides [13,14]. Notably, indigenous Chinese pig breeds like NX possess distinct fat deposition phenotypes, yet the role of gut fungi in regulating such traits remains unexplored. Previous studies on DLY pigs focused on bacterial SCFA producers (e.g., *Firmicutes*, *Bacteroidetes*) [15,16], but neglected fungal contributions. This gap limits holistic understanding of microbial regulation in swine lipid metabolism. Our study addresses this by comparatively analyzing fungal communities in high-fat (NX) and lean (DLY) pigs, hypothesizing that fungi modulate fat deposition via the glucose–SCFA balance.

Thus, this study aims to systematically characterize the gut fungal community structures of NX and DLY pigs to uncover their distinct roles in lipid metabolism. By correlating fungal taxa with host glucose and SCFA profiles, we identify key microbial determinants of fat deposition. Ultimately, we propose a novel regulatory paradigm—the gut fungal-mediated Glucose–SCFAs metabolic axis—to explain the divergent adipogenic phenotypes between these breeds.

## 2. Materials and Methods

### 2.1. Animal Feeding

Ten healthy NX pigs and ten DLY pigs of similar age and body weight (initial weight range: 70 kg ± 2.5 kg), with equal numbers of males and females, were selected. Sample size (*n* = 10/group) was determined by power analysis (α = 0.05, β = 0.8) using pilot data, detecting 25% diversity differences (G*Power v3.1). The experiment lasted 30 days. The 30-day duration was based on established gut microbiota stabilization periods in swine, ensuring detectable metabolic differences while minimizing age-related confounders. Pigs had ad libitum access to water and were fed an equal amount of a basal diet (Table 1, formulated according to NRC 2012) twice daily at 08:00 and 16:00, at a rate of 2.0 kg per pig per day.

### 2.2. Sample Collection

Pigs were fasted for 12 h prior to the end of the experiment. The following morning, 5 mL of blood was collected from the jugular vein. After standing for 30 min, samples were centrifuged at 3000× *g* for 10 min. The supernatant (serum) was aliquoted and stored at −80 °C. Pigs were stunned by electrical stunning followed by exsanguination. Mid-colon contents were aseptically collected, divided into two portions. One portion was mixed with 0.5% H_3_PO_4_, vortexed, and stored at 4 °C for SCFA analysis. The other portion was flash-frozen in liquid nitrogen and stored at −80 °C for fungal DNA extraction.

After slaughter, the head, feet, and viscera were removed, and carcass weight was recorded. Carcasses were split symmetrically along the spine. The left half-carcass was used for body composition analysis. Subcutaneous fat, intermuscular fat, and perirenal fat were dissected, pooled, and weighed (accuracy: 0.1 g). Skeletal muscle was separated, connective tissue and fat were removed, and the lean tissue was weighed.

Fat percentage (%) = (Total fat tissue weight/Carcass weight) × 100,(1)

Lean meat percentage (%) = (Total lean tissue weight/Carcass weight) × 100.(2)

### 2.3. Serum Glucose Analysis (HPLC)

Frozen serum samples were thawed and mixed at 4 °C. A 200 μL aliquot was added to 800 μL of acetonitrile to precipitate proteins, vortexed for 1 min, and centrifuged at 12,000× *g* for 15 min at 4 °C. The supernatant was filtered through a 0.22 μm organic filter membrane before injection. Analysis was performed using an Agilent 1260 HPLC system (Agilent Technologies, Santa Clara, CA, USA) equipped with a ZORBAX-C18 column (4.6 mm × 250 mm, 5 μm). An external standard calibration curve was established using glucose standards (R^2^ ≥ 0.99). Serum glucose concentration (mmol/L) was calculated based on peak area, with the mean value was obtained from triplicate measurements.

### 2.4. Colonic SCFA Analysis (GC-MS)

Frozen colon content samples were thawed, and 50 mg was weighed. One milliliter of pre-treatment solution containing 0.5% H_3_PO_4_ was added. Samples were vortexed for 2 min and centrifuged at 12,000× *g* for 10 min at 4 °C. The supernatant was filtered through a 0.22 μm filter membrane. Analysis was performed using an Agilent 7890B GC system (Agilent Technologies, Santa Clara, CA, USA) coupled with an MS detector, equipped with a DB-FFAP capillary column (30 m × 0.25 mm × 0.25 μm). External standard calibration curves were established using acetate, propionate, and butyrate standards (R^2^ ≥ 0.99). Matrix effects were corrected using 4-methylvaleric acid as the internal standard. SCFA concentrations were calculated based on characteristic ion peak areas and expressed as μg/g wet weight, with the mean value obtained from triplicate measurements.

### 2.5. DNA Extraction and Sequencing

Total DNA was extracted from 200 mg of colon content using the FastDNA^®^ Spin Kit (MP Biomedicals, Shanghai, China) according to the manufacturer’s instructions. The ITS1 (internal transcribed spacer) region, a fungal-specific rRNA marker, was amplified using primers ITS1F and ITS2R. PCR products were verified by 1.5% agarose gel electrophoresis, purified using AMPure XP beads (Beckman Coulter, Brea, CA, USA), and stored at −20 °C. Purified amplicons were used to construct TruSeq DNA PCR-Free libraries and sequenced (paired-end, 2 × 250 bp) on an Illumina NovaSeq 6000 platform (Illumina, Inc., San Diego, CA, USA). Raw sequence data were processed within QIIME2 (2023.9): sequences with Q-score < 30 were filtered, retaining >5000 reads/sample. Primers were trimmed using cutadapt, and denoising, chimera removal, and generation of the OTU table were performed using DADA2 (v1.26.0).

### 2.6. Bioinformatics Analysis

Alpha diversity indices (Shannon index and Chao1 index) were calculated based on the OTU table. Beta diversity differences were visualized using Principal Coordinates Analysis (PCoA) based on Bray–Curtis dissimilarity matrices. OTUs were taxonomically classified, and relative abundances (%) at the genus level were calculated within each sample. Linear Discriminant Analysis Effect Size (LEfSe) was employed to identify fungal taxa differentially abundant between groups. Fungal functional guilds were predicted using the FUNGuild database, and results with a confidence rating of “Probable” or higher were retained for analysis.

### 2.7. Statistical Analysis

All data were tested for normality (Shapiro–Wilk test) and homogeneity of variance (Levene’s test). Non-normally distributed data were log-transformed prior to analysis. Differences between groups (NX vs. DLY) for serum glucose, SCFA concentrations, and alpha diversity indices (Shannon, Chao1) were assessed using independent samples *t*-tests (for normally distributed data with homoscedasticity) or Mann–Whitney U tests (for non-normally distributed data or heteroscedasticity). Beta diversity differences based on Bray–Curtis distance matrices were evaluated using Permutational Multivariate Analysis of Variance (PERMANOVA) with 999 permutations. For LEfSe analysis, taxa with a Linear Discriminant Analysis (LDA) score > 2.5 and a Kruskal–Wallis test *p*-value < 0.05 were considered significant, and the Benjamini–Hochberg procedure was used to control the False Discovery Rate (FDR) for multiple hypothesis testing. Differences in predicted FUNGuild functional guild abundances between groups were assessed using the Kruskal–Wallis test (*p* < 0.05). Spearman’s rank correlation analysis (|r| > 0.5, *p* < 0.05) was performed to assess relationships between significantly different fungal taxa (or functional guilds) and serum glucose or intestinal SCFA concentrations.

Statistical analyses were performed using R (4.3.2). Data visualization was conducted using the Python Seaborn (0.12.2) and Matplotlib (3.7.1) libraries. Microbiome community data were processed using QIIME2 (2023.9). Statistical significance was set at *p* < 0.05, and trends were noted for 0.05 ≤ *p* < 0.10.

## 3. Results

### 3.1. Phenotypic Differences

**Figure 1:**
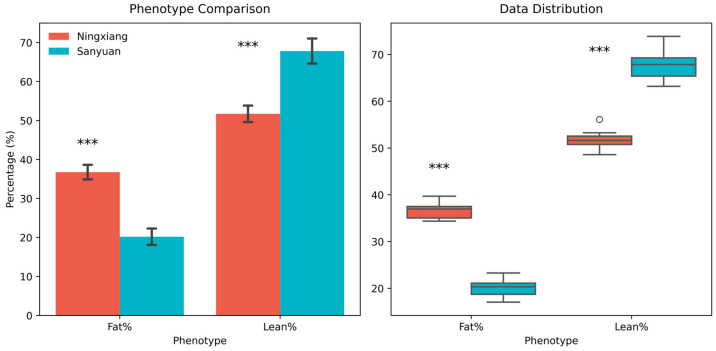
Comparison of body composition and metabolite concentrations between NX and DLY pigs. NX pigs had significantly higher fat percentage (36.8% ± 1.9%) and lower lean meat percentage (51.7% ± 2.1%) than DLY pigs (20.2% ± 2.1% and 67.8% ± 3.2%, respectively; *p* < 0.001). Total colonic SCFAs (acetate, propionate, butyrate) were 38.8% lower, while serum glucose was 38.1% higher in NX pigs (*p* < 0.001). Error bars represent mean ± SD; *** denotes *p* < 0.001; Independent samples *t*-test.

NX pigs exhibited significantly higher fat percentage (36.8% ± 1.9%) and lower lean meat percentage (51.7% ± 2.1%) compared to DLY pigs (fat percentage: 20.2% ± 2.1%; lean meat percentage: 67.8% ± 3.2%) (*p* < 0.001) (Figure 1).

**Figure 2:**
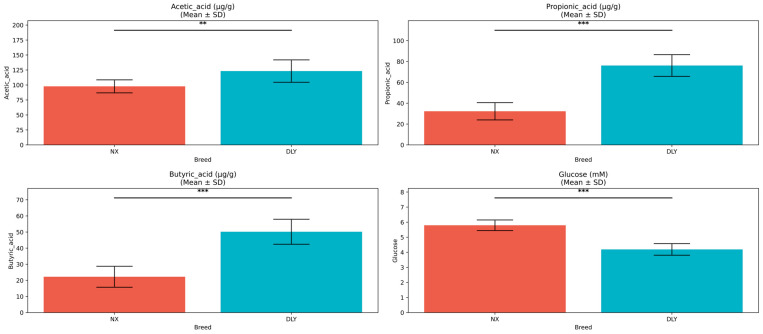
Differences in colonic SCFAs and serum glucose between NX and DLY pigs. Colonic SCFA concentrations were significantly lower in NX pigs (acetate: 91 ± 15 vs. 128 ± 17 μg/g; propionate: 41 ± 11 vs. 79 ± 12 μg/g; butyrate: 24 ± 8 vs. 48 ± 11 μg/g; *p* < 0.05), while serum glucose was significantly elevated (5.8 ± 0.4 vs. 4.2 ± 0.3 mmol/L, *p* < 0.001). Error bars represent mean ± SD; ** denotes *p* < 0.01, *** denotes *p* < 0.001; Mann–Whitney U test.

Further analysis revealed lower colonic SCFA concentrations in NX pigs, with acetate being the most abundant SCFA (91 ± 15 vs. 128 ± 17 μg/g, *p* = 0.015). Propionate (41 ± 11 vs. 79 ± 12 μg/g, *p* < 0.001) and butyrate (24 ± 8 vs. 48 ± 11 μg/g, *p* < 0.001) showed highly significant reductions. Conversely, serum glucose concentration was markedly elevated in NX pigs (5.8 ± 0.4 vs. 4.2 ± 0.3 mmol/L, *p* < 0.001) (Figure 2). These results suggest divergent metabolic pathways for SCFAs and glucose between the two breeds.

### 3.2. Associations Between Body Composition and Metabolites

**Figure 3:**
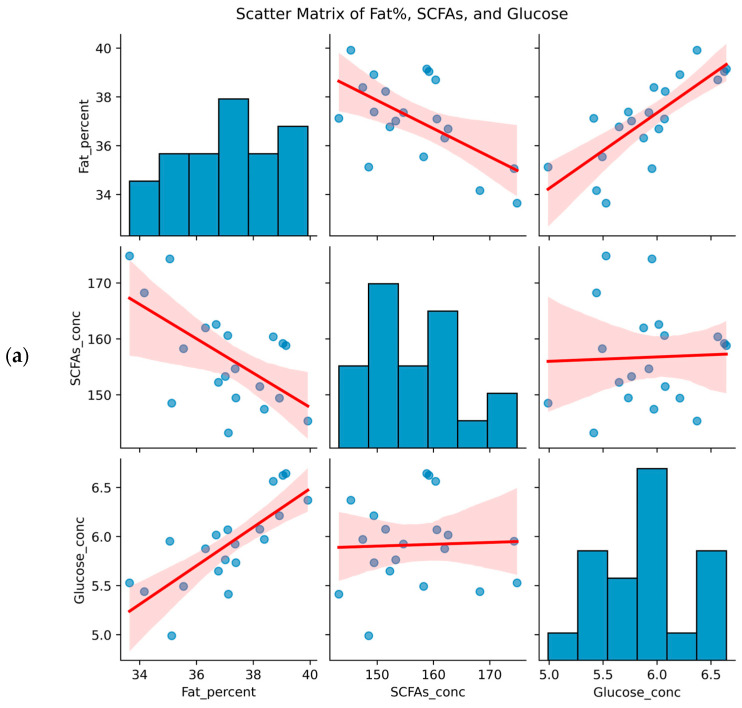

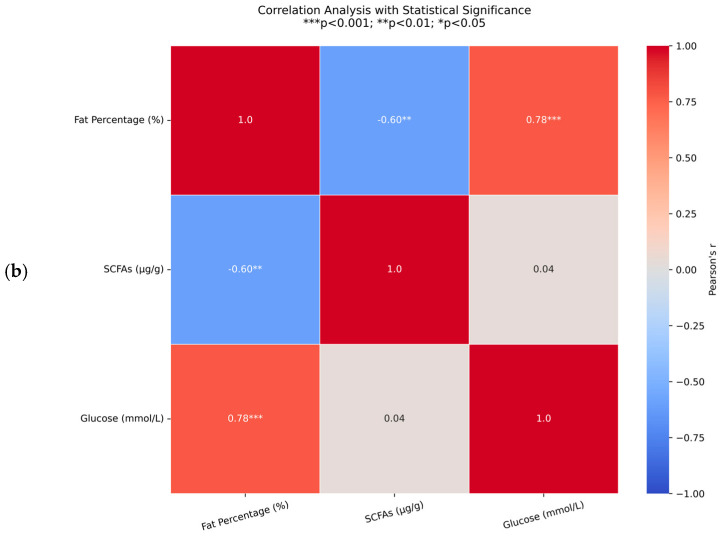
Scatterplot matrix and regulatory network between body composition and metabolites. (**a**) Diagonal: Kernel density plots of variable distributions; Off-diagonal: Scatterplots with Spearman correlations (|r| > 0.5, *p* < 0.05). Blue lines = regression fits. (**b**) In NX pigs, fat percentage correlated positively with serum glucose (r = 0.78, *p* = 0.0056) and negatively with total colonic SCFAs (r = −0.60, *p* < 0.001). No significant associations were observed between SCFAs and glucose.

Scatterplot matrices (Figure 3a) revealed complex relationships in NX and DLY pigs: fat percentage showed a significant positive correlation with serum glucose (r = 0.78, *p* = 0.0056, Figure 3b) and a significant negative correlation with total colonic SCFAs (r = −0.60, *p* < 0.001, Figure 3b). The weak association between SCFAs and glucose suggests relatively independent regulatory pathways. This indicates that glucose metabolism may synergistically drive high fat deposition in NX pigs, while SCFAs—as key substrates for intestinal energy metabolism—likely promote fat consumption by inhibiting adipocyte differentiation or stimulating lipolysis.

### 3.3. Alpha and Beta Diversity Analysis

**Figure 4:**
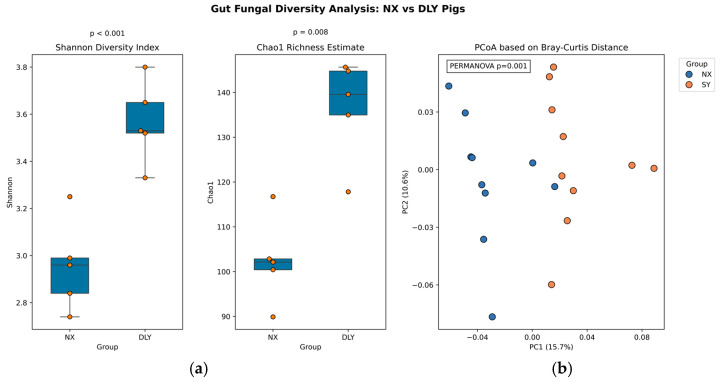
Alpha and beta diversity analysis of gut fungal community. (**a**) Shannon index: 3.5 ± 0.2 vs. 2.8 ± 0.3; Chao1 index: 140 ± 13 vs. 97 ± 14; *p* < 0.05; (**b**) PERMANOVA R^2^ = 0.18, *p* = 0.002; PC1 and PC2 explain 28.3% and 19.6% of variation, respectively.

NX pigs harbored significantly higher gut fungal alpha diversity (Shannon index: 3.5 ± 0.2 vs. 2.8 ± 0.3; Chao1 index: 140 ± 13 vs. 97 ± 14; *p* < 0.05; Figure 4a). Beta diversity analysis (PCoA based on Bray–Curtis distances) revealed clear separation between breeds (PERMANOVA R^2^ = 0.18, *p* = 0.002), indicating distinct community structures (Figure 4b). While bacterial communities (e.g., *Firmicutes*/*Bacteroidetes* ratio) were not sequenced here, prior studies confirm their dominant role in SCFA production [15,16]. Our fungal-focused approach reveals previously overlooked regulatory pathways.

### 3.4. Fungal Compositional Differences

**Figure 5:**
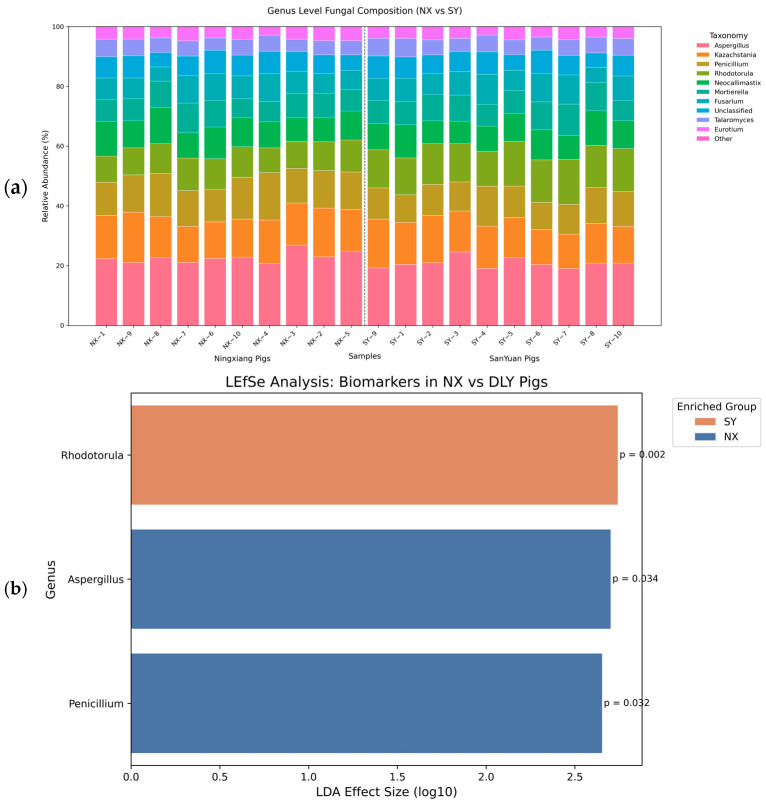
Genus-level composition and LEfSe analysis of gut fungi in NX and DLY pigs. (**a**) NX pigs showed enriched *Aspergillus* (22.2% ± 1.8%) and *Penicillium* (12.5% ± 1.2%; *p* < 0.01), while DLY pigs had higher *Rhodotorula* abundance (14.3% ± 1.5% vs. 9.6% ± 1.4%; *p* < 0.01). Bars show relative abundance of top 10 genera. *p* < 0.01, Kruskal–Wallis test; (**b**) LEfSe identified NX-associated genera *Aspergillus* (LDA = 2.70, *p* = 0.01) and *Penicillium* (LDA = 2.65, *p* = 0.006), and DLY-associated *Rhodotorula* (LDA = 2.74, *p* < 0.001). Bars show taxa with LDA > 2.5.

Taxonomic analysis showed significant enrichment of *Aspergillus* (relative abundance: 22.2% ± 1.8% vs. 20.2% ± 1.6%) and *Penicillium* (12.5% ± 1.2% vs. 10.6% ± 1.4%) in NX pigs (*p* < 0.01), while *Rhodotorula* dominated in DLY pigs (14.3% ± 1.5% vs. 9.6% ± 1.4%, *p* < 0.01) (Figure 5a). LEfSe analysis identified *Aspergillus* (LDA = 2.70, *p* = 0.01) and *Penicillium* (LDA = 2.65, *p* = 0.006) as discriminative taxa for NX pigs, and *Rhodotorula* for DLY pigs (LDA = 2.74, *p* < 0.001) (Figure 5b). These compositional differences suggest evolutionary divergence in energy metabolism strategies.

### 3.5. Fungal Functional Prediction and Metabolic Correlations

**Figure 6:**
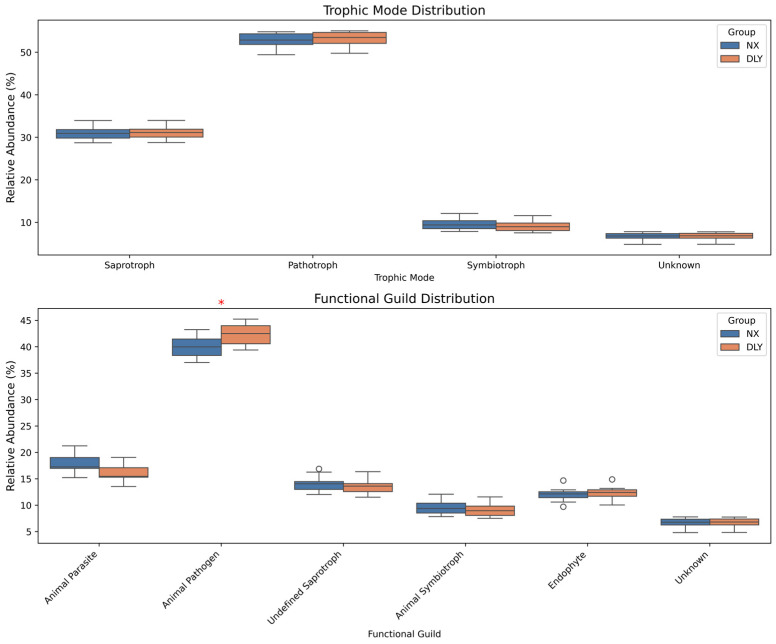
Intergroup differences in FUNGuild functional guilds. DLY pigs showed significantly higher “Animal Pathogen” guild abundance (* denotes *p* < 0.05). No guilds were enriched in NX pigs.

FUNGuild annotation revealed similar nutritional modes but significant differences in specific functional guilds. DLY pigs had significantly higher abundance of the “Animal Pathogen” guild (*p* < 0.05) (Figure 6), suggesting greater pathogenic colonization risk. Conversely, NX pigs may possess enhanced resistance to pathogens.

**Figure 7:**
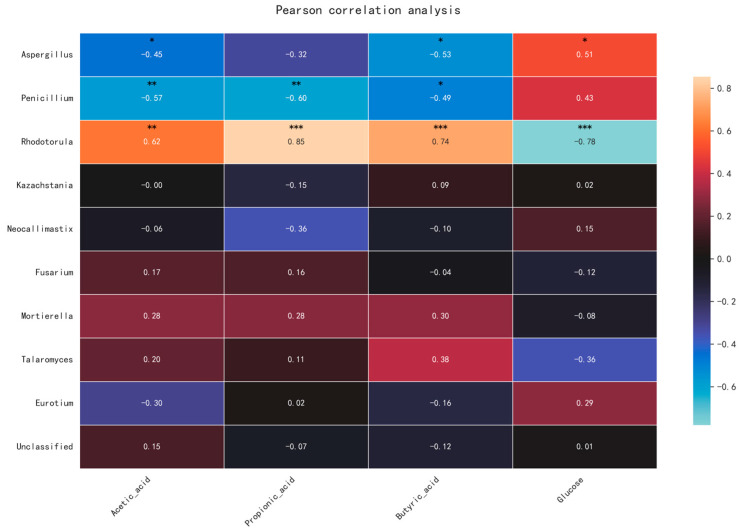
Heatmap of Spearman correlations between fungal genera and metabolites. In NX pigs, *Aspergillus* positively correlated with serum glucose (r = 0.51, *p* < 0.05) and negatively with SCFAs (*Penicillium*-propionate: r = −0.60, *p* < 0.01). In DLY pigs, *Rhodotorula* positively correlated with SCFAs (propionate: r = 0.85, *p* < 0.001). Color gradient indicates correlation coefficient (|r| > 0.5, *p* < 0.05). * denotes *p* < 0.05, ** denotes *p* < 0.01, *** denotes *p* < 0.001.

Correlation analysis showed that the dominant NX genera (*Aspergillus*, *Penicillium*) strongly correlated negatively with SCFAs (*Penicillium*-propionate: r = −0.72, *p* < 0.01) and positively with serum glucose (*Aspergillus*: r = 0.51, *p* < 0.01). In DLY pigs, *Rhodotorula* correlated positively with SCFAs (propionate: r = 0.85, *p* < 0.001) and negatively with glucose (r = −0.78, *p* < 0.001) (Figure 7), indicating co-evolution between fungal function and host metabolic phenotype.

## 4. Discussion

This study reveals a novel mechanism by which the gut fungal community regulates fat deposition in NX pigs through microbial metabolites. Notably, NX pigs exhibited a significantly higher fat deposition rate compared to DLY pigs. Their serum glucose levels were elevated by 38.1%, while the concentration of total SCFAs in colonic contents was 38.8% lower than in DLY pigs. Specifically, acetate, propionate, and butyrate decreased by 29.0%, 48.1%, and 50.0%, respectively. Further analysis revealed a significant negative correlation between the fat rate and total colonic SCFAs concentration, and a significant positive correlation with serum glucose concentration. This association suggests that the high fat deposition in NX pigs may be closely linked to the accumulation of glucose in their serum. Furthermore, butyrate, a key substrate in energy metabolism, might drive fat consumption by inhibiting adipocyte differentiation or promoting lipolysis.

Analysis of ITS sequencing revealed a significant specificity in the gut fungal community of NX pigs: the relative abundance of *Aspergillus* and *Penicillium* increased compared to DLY pigs, while the abundance of *Rhodotorula* decreased. Studies indicate that *Penicillium* spp. can secrete glucose oxidase, leading to increased glucose consumption in the local microenvironment and consequently elevated circulating blood glucose levels [17,18]. The cellulase systems of *Aspergillus* spp. may enhance polysaccharide breakdown within the host gut, increasing glucose bioavailability [19,20]. Our study also found a strong positive correlation between the abundance of the *Aspergillus*/*Penicillium* complex and serum glucose levels, suggesting that these gut fungi promote fat deposition, at least partly, through a monosaccharide supply regulatory axis. Mechanistically, serum glucose likely serves as a substrate for de novo lipogenesis in the liver. It may upregulate the expression of key enzymes such as fatty acid synthase and acetyl-CoA carboxylase via the insulin signaling pathway [21,22], thereby explaining the characteristic subcutaneous fat deposition in NX pigs. This proposed glucose-driven mechanism aligns with findings in Jinhua pigs, where an *Aspergillus*-enriched microbiota correlated with elevated lipogenesis [23].

Complementing this glucose-mediated pathway, our data also implicate reduced SCFA biosynthesis, linked to lower *Rhodotorula* abundance, in promoting adiposity. We observed a significant positive correlation between *Rhodotorula* abundance and colonic SCFAs concentration. Although existing research does not directly establish a link between *Rhodotorula* abundance and gut SCFA levels, it may influence SCFA production through interactions with other gut microbes. For instance, an in vitro study evaluated the nutritional quality of microbial samples from bacteria, filamentous fungi, and yeasts, including a yeast strain (*Rhodotorula*) [24]. This study measured in vitro protein digestibility and carbohydrate colonic fermentation, including SCFA production. Thus, the reduction in *Rhodotorula* observed in NX pigs may impair gut SCFA biosynthesis, particularly butyrate. As a crucial regulator of lipid metabolism, butyrate can inhibit adipocyte differentiation by activating AMP-activated protein kinase phosphorylation [25,26,27]. The consequent reduction in butyrate concentration in NX pigs may therefore alleviate the inhibition of adipocyte differentiation, creating a microenvironment conducive to lipid deposition. Collectively, this ‘Glucose–SCFAs axis’ mediated by gut fungi provides a dual mechanism promoting fat deposition in NX pigs, complementing known bacterial contributions such as acetate production by *Bacteroides* spp. for fat storage and butyrate-induced leptin expression by *Roseburia* [28,29].

Notably, this study challenges the traditional view in prior research that emphasizes the dominant role of bacterial SCFAs in lipid metabolism [30], uncovering the potential contribution of gut fungi in monogastric animals. Unlike ruminants where fungi degrade fiber in foreguts [31,32], porcine fungi directly modulate host glucose metabolism—a distinction arising from monogastric physiology and high-starch diets. This species-specific mechanism necessitates breed-customized microbial modulation strategies for the livestock industry. Practically, manipulating gut fungi offers targeted solutions: for NX pigs, reducing *Aspergillus*/*Penicillium* (e.g., via antifungal feed additives) may decrease excessive fat deposition; for DLY pigs, supplementing *Rhodotorula* could enhance intramuscular fat for superior meat quality. This approach is particularly valuable for indigenous breeds—optimizing NX pigs’ pathogen resistance with fat modulation could maximize their use in specialty pork production. Industrial applications could include balancing lipogenesis in fatty breeds (*Rhodotorula* enhancement) or improving flavor in lean-type pigs (targeted *Aspergillus*/*Penicillium* use). However, while bioinformatic correlations link fungal metabolism to fat deposition, direct causality requires validation through fungal isolation, culture, and in vitro studies.

## 5. Conclusions

The high fat rate in NX pigs is associated with glucose enrichment and the dominance of cellulose-degrading fungi (*Aspergillus*/*Penicillium*), whereas DLY pigs’ leanness associates with *Rhodotorula*-linked SCFA production. Targeted modulation of the gut fungal community could potentially optimize pig production performance and provide a theoretical basis for the utilization of indigenous breed resources. Targeting key fungi (e.g., probiotics/antifungals) could fine-tune fat/lean ratios in swine production, improving economic efficiency and product diversity.

## Figures and Tables

**Table 1:** Composition and nutrient levels of diets (air-dry basis) %.

Items	Content
Ingredient Composition ^†^	
Corn	60.0
Soybean meal	25.0
Wheat bran	8.0
CaHPO_4_	2.0
Limestone	1.5
NaCl	0.3
Premix ^1^	3.0
Soybean Oil	0.2
Total	100.0
Nutrient levels	
DE, MJ/kg	14.28
CP	16.94
CF	4.15
EE	4.72
Ca	0.68
Total P	0.51

^†^ Identical diet fed to both breeds to eliminate dietary confounding. ^1^ The basal diet was supplemented with 3% premix per ton, providing vitamins (A, D, E, K, B-complex), trace minerals (Fe, Cu, Zn, Mn, Se, I), and antioxidants to meet NRC (2012) nutrient recommendations.

## Data Availability

The data presented in this study are available on request from the corresponding author.

## References

[1] Bordbar F., Mohammadabadi M., Jensen J., Xu L., Li J., Zhang L. (2022). Identification of Candidate Genes Regulating Carcass Depth and Hind Leg Circumference in Simmental Beef Cattle Using Illumina Bovine Beadchip and Next-Generation Sequencing Analyses. Animals.

[2] Mohammadabadi M., Bordbar F., Jensen J., Du M., Guo W. (2021). Key Genes Regulating Skeletal Muscle Development and Growth in Farm Animals. Animals.

[3] He J., Zhang P., Shen L., Niu L., Tan Y., Chen L., Zhao Y., Bai L., Hao X., Li X. (2020). Short-Chain Fatty Acids and Their Association with Signalling Pathways in Inflammation, Glucose and Lipid Metabolism. Int. J. Mol. Sci..

[4] Portincasa P., Bonfrate L., Vacca M., De Angelis M., Farella I., Lanza E., Khalil M., Wang D.Q.H., Sperandio M., Di Ciaula A. (2022). Gut Microbiota and Short Chain Fatty Acids: Implications in Glucose Homeostasis. Int. J. Mol. Sci..

[5] Lu Y., Fan C., Li P., Lu Y., Chang X., Qi K. (2016). Short Chain Fatty Acids Prevent High-fat-diet-induced Obesity in Mice by Regulating G Protein-coupled Receptors and Gut Microbiota. Sci. Rep..

[6] Den Besten G., Bleeker A., Gerding A., van Eunen K., Havinga R., van Dijk T.H., Oosterveer M.H., Jonker J.W., Groen A.K., Reijngoud D.J. (2015). Short-Chain Fatty Acids Protect Against High-Fat Diet–Induced Obesity via a PPARγ-Dependent Switch From Lipogenesis to Fat Oxidation. Diabetes.

[7] Giovanni T., Vincenzo C. (2024). What are the common downstream molecular events between alcoholic and nonalcoholic fatty liver?. Lipids Health Dis..

[8] Milton-Laskibar I., Marcos-Zambrano L.J., Gómez-Zorita S., de Santa Pau E.C., Fernández-Quintela A., Martínez J.A., Portillo M.P. (2022). Involvement of microbiota and short-chain fatty acids on non-alcoholic steatohepatitis when induced by feeding a hypercaloric diet rich in saturated fat and fructose. Gut Microbiome.

[9] Shi K., Zhang C., Duan C.J., Tan C., Zhang S., Wu J., Liu W.-F., Su T. (2013). The correlation of adipocyte size, serum insulin and leptin levels in high-fat-induced obesity mouse. China J. Mod. Med..

[10] Schaefer E.J., Gleason J.A., Dansinger M.L. (2009). Dietary Fructose and Glucose Differentially Affect Lipid and Glucose Homeostasis. J. Nutr..

[11] Deehan E.C., Mocanu V., Madsen K.L. (2024). Effects of dietary fibre on metabolic health and obesity. Nat. Rev. Gastroenterol. Hepatol..

[12] Wang L.Y., He L.H., Xu L.J., Li S.B. (2024). Short-chain fatty acids: Bridges between diet, gut microbiota, and health. J. Gastroenterol. Hepatol..

[13] Khosravi C., Benocci T., Battaglia E., Benoit I., de Vries R.P. (2015). Sugar Catabolism in Aspergillus and Other Fungi Related to the Utilization of Plant Biomass. Adv. Appl. Microbiol..

[14] Glass N.L., Schmoll M., Cate J.H., Coradetti S. (2013). Plant Cell Wall Deconstruction by Ascomycete Fungi. Annu. Rev. Microbiol..

[15] Yang L., Bian G., Su Y., Zhu W. (2014). Comparison of Faecal Microbial Community of Lantang, Bama, Erhualian, Meishan, Xiaomeishan, Duroc, Landrace, and Yorkshire Sows. Asian-Australas. J. Anim. Sci..

[16] Xiao Y., Li K., Xiang Y., Zhou W., Gui G., Yang H. (2017). The fecal microbiota composition of boar Duroc, Yorkshire, Landrace and Hampshire pigs. Asian-Australas. J. Anim. Sci..

[17] Doolotkeldieva T.D., Bobusheva S.T. (2011). Screening of Wild-Type Fungal Isolates for Cellulolytic Activity. Microbiol. Insights.

[18] Simpson C., Jordaan J., Gardiner N.S., Whiteley C. (2007). Isolation, purification and characterization of a novel glucose oxidase from *Penicillium* sp. CBS 120262 optimally active at neutral pH. Protein Expr. Purif..

[19] Devi A., Singh A., Kothari R. (2024). Fungi based valorization of wheat straw and rice straw for cellulase and xylanase production. Sustain. Chem. Environ..

[20] Naher L., Fatin S.N., Sheikh M.A.H., Azeez L.A., Siddiquee S., Zain N.M., Karim S.M.R. (2021). Cellulase Enzyme Production from Filamentous Fungi Trichoderma reesei and Aspergillus awamori in Submerged Fermentation with Rice Straw. J. Fungi.

[21] Clarke S.D., Nakamura M.T. (2013). Lipids|Fatty Acid Structure and Synthesis. Encyclopedia of Biological Chemistry III.

[22] Scoditti E., Sabatini S., Carli F., Gastaldelli A. (2024). Hepatic glucose metabolism in the steatotic liver. Nat. Rev. Gastroenterol. Hepatol..

[23] Yang H., Xiang Y., Robinson K., Wang J., Zhang G., Zhao J., Xiao Y. (2018). Gut Microbiota Is a Major Contributor to Adiposity in Pigs. Front. Microbiol..

[24] Hernández-Almanza A., Cesar Montanez J., Aguilar-González M.A., Martínez-Ávila C., Rodríguez-Herrera R., Aguilar C.N. (2014). Rhodotorula glutinis as source of pigments and metabolites for food industry. Food Biosci..

[25] Li H., Liu S., Chen H., Zhou L., Chen B., Wang M., Zhang D., Han T.L., Zhang H. (2024). Gut dysbiosis contributes to SCFAs reduction-associated adipose tissue macrophage polarization in gestational diabetes mellitus. Life Sci..

[26] Jeon T., Hwang S.G., Hirai S., Matsui T., Yano H., Kawada T., Lim B.O., Park D.K. (2004). Red yeast rice extracts suppress adipogenesis by down-regulating adipogenic transcription factors and gene expression in 3T3-L1 cells. Life Sci..

[27] Lan Y., Sun Q., Ma Z., Peng J., Zhang M., Wang C., Zhang X., Yan X., Chang L., Hou X. (2022). Seabuckthorn polysaccharide ameliorates high-fat diet-induced obesity by gut microbiota-SCFAs-liver axis. Food Funct..

[28] Si X., Shang W., Zhou Z., Strappe P., Wang B., Bird A., Blanchard C. (2018). Gut Microbiome-Induced Shift of Acetate to Butyrate Positively Manages Dysbiosis in High Fat Diet. Mol. Nutr. Food Res..

[29] Tamanai-Shacoori Z., Smida I., Bousarghin L., Loreal O., Meuric V., Fong S.B., Bonnaure-Mallet M., Jolivet-Gougeon A. (2017). *Roseburia* spp.: A Marker of Health?. Future Microbiol..

[30] Zhou H., Yu B., Sun J., Liu Z., Chen H., Ge L., Chen D. (2020). Short-chain Fatty Acids can Improve Lipid and Glucose Metabolism Independently of the Gut Microbiota. J. Anim. Sci. Biotechnol..

[31] Bhagat N.R., Kumar S., Kumari R., Bharti V.K. (2023). A Review on Rumen Anaerobic Fungi: Current Understanding on Carbohydrate Fermentation and Roughages Digestion in Ruminants. Appl. Biochem. Microbiol..

[32] Thapa S., Mishra J., Arora N., Mishra P., Li H., O′ Hair J., Bhatti S., Zhou S. (2020). Microbial cellulolytic enzymes: Diversity and biotechnology with reference to lignocellulosic biomass degradation. Rev. Environ. Sci. Bio/Technol..

